# Characteristics of gut microbiota in patients with primary Sjögren’s syndrome in Northern China

**DOI:** 10.1371/journal.pone.0277270

**Published:** 2022-11-10

**Authors:** Yuyuan Li, Zhi Li, Wenying Sun, Meiling Wang, Ming Li

**Affiliations:** 1 Advanced Institute for Medical Sciences, Dalian Medical University, Dalian, China; 2 Department of Clinical Laboratory, Dalian Municipal Central Hospital, Dalian, China; 3 College of Basic Medical Science, Dalian Medical University, Dalian, China; University of Minnesota Twin Cities, UNITED STATES

## Abstract

This study analyzes and compares the structure and diversity of gut microbiota in patients with primary Sjögren’s syndrome (pSS) in Northern China to healthy individuals to identify clinical features associated with dysbiosis. We included 60 Chinese pSS patients and 50 age- and gender-matched healthy controls. DNA was extracted from stool samples and subjected to 16S ribosomal RNA gene analysis (V3-V4) for intestinal dysbiosis. In addition, patients were examined for laboratory and serological pSS features. A Spearman’s correlation analysis was performed to assess correlations between individual bacteria taxa and clinical characteristics. The alpha-diversity (Chao1 and Shannon Index) and beta-diversity (unweighted UniFrac distances) of the gut microbiota differed significantly between pSS patients and healthy controls. Further analysis showed that several gut opportunistic pathogens (*Bacteroides*, *Megamonas*, and *Veillonella*) were significantly more abundant in pSS patients and positively correlated with their clinical indicators. In contrast, some probiotic genera (*Collinsella*, unidentified_*Ruminococcaceae*, *Romboutsia*, and *Dorea*) were significantly decreased in pSS patients and negatively correlated with their clinical indicators. Therefore, pSS patients in Northern China showed a dysbiotic intestinal microbiome enriched for potentially pathogenic genera that might be associated with autoimmune disease.

## Introduction

Primary Sjögren’s syndrome (pSS) is a systemic autoimmune disease with a worldwide prevalence of 0.01%-0.09%, characterized by the infiltration of leukocytes into the exocrine glands, particularly the salivary and lachrymal glands [1, 2]. It is much more common in females than in males, particularly in middle-aged women [3]. The pSS prevalence rate in China is approximately 0.33%-0.77%, depending on the diagnostic criteria used, higher than in other countries [4–6].

Its pathogenesis and etiology are poorly understood. Evidence suggests that dysbiosis of the gut microbiome contributes to the pathogenesis of several autoimmune diseases, such as inflammatory bowel disease (IBD), systemic lupus erythematosus (SLE), and rheumatoid arthritis (RA) [7–9]. Dysbiosis of the gut microbiome has also been implicated in pSS. The pilot study by de Paiva et al. [10] showed that gut dysbiosis exacerbated experimental pSS in mice and correlated with disease severity. Similarly, intestinal dysbiosis has been reported in pSS patients, associated with clinical and laboratory markers of disease activity and signs of gastrointestinal inflammation [11]. However, they did not consider or report significant differences in microbiota. Wang et al. [12] reported that the intestinal microbiota of pSS patients differed significantly among high active disease, low active disease, and control groups, especially the *Streptococcu* genus. Recently, Yang et al. [13] showed that the pSS gut microbiota is characterized by increased pro-inflammatory and decreased anti-inflammatory microbes. However, the correlation between differences in microbiota and clinical indicators remain unknown.

To explore the intestinal microbial balance of pSS patients in Northern China, we extracted total DNA from the fresh feces of 60 patients with RA and 50 healthy controls for 16S ribosomal RNA gene sequencing. We found differences in the gut microbiota. In addition, we correlated the gut microbiota of pSS patients with candidate biomarkers, including autoantibodies (antinuclear [ANA], anti-Ro52, anti-Ro/SSA, and anti-La/SSB) [14], rheumatoid factor (RF), C-reactive protein (CRP), immunoglobulins (Ig: IgG, IgA, IgM, and IgE), and complement components 3 (C3) and 4 (C4).

## Materials and methods

### Subject recruitment and clinical characteristics

We recruited 60 pSS patients aged 36–84 years from Dalian Municipal Central Hospital (Dalian, China) between January 2021 and February 2022. All pSS patients met the 2002 American-European Consensus group classification criteria [15] and the American College of Rheumatology/European League Against Rheumatism classification criteria [16]. None had used local or systemic antibiotics or probiotics in the last three months. Patients with secondary Sjögren’s syndrome, other autoimmune diseases, or gastrointestinal tract disorders were excluded. In addition, 50 age- and sex-matched healthy control individuals that met none of the pSS diagnostic criteria were enrolled in this study. The exclusion criteria for the healthy controls were: diagnosis of gut disease and use of antibiotics or probiotics within the last 3 months prior. This study was approved by the Ethics Committees of Dalian Municipal Central Hospital (Dalian, China; YN2021-002-01). All participants provided written informed consent before participation in this study.

### Sampling and laboratory analyses

Serum samples were collected from all study participants and stored at -80°C until analysis. Fecal samples were obtained from 41 pSS patients and 44 healthy controls. Serum ANA was measured by the indirect immunofluorescence technique on a HEp-2 cell substrate. Serum anti-Ro52, anti-Ro/SSA, and anti-La/SSB antibody levels were determined using an enzyme-linked immunosorbent assay kit (Quintiles Laboratories North America; Marietta, GA, USA). An immunoturbidimetric assay was used to determine the serum levels of RF, CRP, IgG, IgA, IgM, IgE, C3, and C4 (Beckman Coulter, CA, USA). All analyses were performed at Dalian Municipal Central Hospital.

### Genomic DNA extraction and gut microbiota sequencing

DNA was extracted from 200 mg (fresh weight) of each fecal sample using the QIAamp DNA stool Mini kit (Qiagen; Hilden, Germany) according to the manufacturer’s protocol. DNA concentration was measured and its purity was confirmed with a Nanodrop2000 (Thermo Fisher Scientific; Wilmington, CA, USA). The gut bacterial V3-V4 region was amplified using primers 341F (5’-CCTAYGGGRBGCASCAG-3’) and 806R (5’-GGACTACNNGGGTATCTAAT-3’). PCR reactions were performed with an initial denaturation step of 98°C for 2 min, 25 cycles of denaturation at 98°C for 15 s, annealing at 55°C for 30 s, and extension at 72°C for 30 s, followed by a final extension step of 72°C for 5 min before being held at 4°C. PCR amplicon sequencing was performed using the Illumina HiSeq platform at Novogene Bioinformatics Technology Co., Ltd. (Beijing, China).

### Bioinformatic analysis

Sequence data analyses were mainly performed using QIIME (v.1.9.1) and R software packages (v.2.15.3). For operational taxonomic unit-based analysis, sequences were clustered using Uparse (v.7.0.1001) with a similarity cutoff of 97%. Community richness and diversity (alpha diversity) analysis were measured by Chao1 and Shannon index. Beta diversity was measured using principal coordinate analysis (PCoA) with unweighted UniFrac analysis in the R software. The linear discriminant analysis (LDA) effect size (LEfSe) analysis was used to identify differentially abundant taxa across two groups. Correlations among variables were assessed using Spearman’s rank correlation coefficient.

### Statistical analysis

Continuous and normally distributed variables are presented as arithmetic means and standard error of the mean (SEM). The significance of data differences (*P*≤0.05) was assessed using a nonparametric t-test in GraphPad Prism 7 (Graph Pad Software; La Jolla, CA, USA). The statistical analysis of beta diversity used the nonparametric “Adonis” method in the “vegan” package of the QIIME-incorporated version of the R software. Correlation analyses used Spearman’s rank correlation tests. Statistical analyses were performed in SPSS v.9.0 (SPSS Inc.; Chicago, IL, USA).

### Accession number

The sequence data associated with this study are deposited in the NCBI Sequence Read Archive with the accession number PRJNA856785.

## Results

### Clinical characteristics

This study enrolled 110 participants. **Table 1** lists the demographic, clinical, and laboratory characteristics of the pSS patients and healthy controls. The pSS patients (n = 60) had a mean age of 59.37±1.25 years, and 95% were female. The healthy controls (n = 50) had a mean age of 60.0±1.44 years, and 90% were female. Age and sex did not differ significantly between the pSS and control participants (*P*>0.05). In addition, marital status, smoking status, education, occupation, and diet (including vegetarianism) did not differ significantly between the pSS and control participants (*P*>0.05; **S1 Table**). ANA, anti-SSA, anti-SSB, and anti-Ro52 positivity in pSS patients were 95.0%, 68.3%, 26.7%, 78.3%, respectively. In addition, pSS patients had significantly higher RF (*P* = 0.0110), IgG (*P*<0.0001), IgA (*P* = 0.0006), and C3 (*P* = 0.0002) levels than controls.

**Table 1:** The demographic and clinical characteristics of participants.

	pSS patients (n = 60)	Healthy control (n = 50)	*P*
Sex (female), n (%)	57 (95.0%)	45 (90.0%)	0.3190
Age (mean±SEM), years	59.37±1.25	60.00±1.44	0.7400
Disease duration (mean±SEM), months	75.48±10.31	-	
ANA (positive), n (%)	55 (95.0%)	-	
Anti-SSA (positive), n (%)	41 (68.3%)	-	
Anti-SSB (positive), n (%)	16 (26.7%)	-	
Anti-Ro52 (positive), n (%)	47 (78.3%)	-	
RF (mean±SEM), IU/mL	71.63±20.62	12.54±2.62	0.0110*
CRP (mean±SEM), mg/L	4.66±1.31	5.37±1.34	0.7080
IgG (mean±SEM), IU/mL	16.39±0.80	10.61±0.31	<0.0001****
IgA (mean±SEM), IU/mL	3.06±0.26	1.98±0.11	0.0006***
IgM (mean±SEM), IU/mL	1.05±0.07	0.88±0.06	0.0990
IgE (mean±SEM), IU/mL	54.68±9.62	59.25±10.85	0.7500
C3 (mean±SEM), g/mL	0.87±0.02	1.10±0.02	0.0002***
C4 (mean±SEM), g/mL	0.19±0.01	0.72±0.47	0.2180

Key

*, *P*<0.05

***, *P*<0.001

****, *P*<0.0001.

### Comparison of the diversity of gut bacterial communities between pSS patients and healthy controls

**Fig 1:**
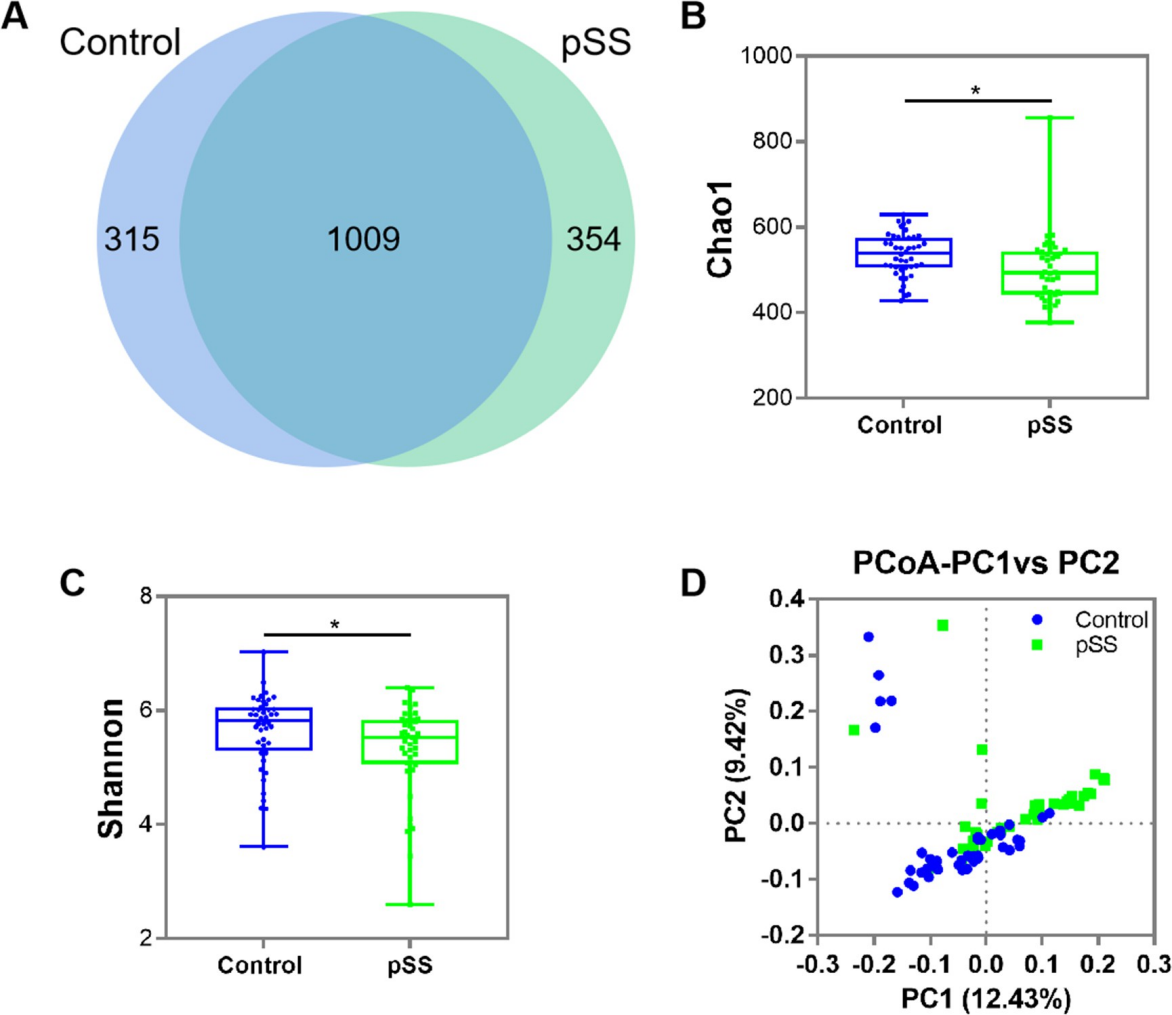
Comparison of gut microbial diversity in the pSS and control groups. (**A**) Venn diagram showing the overlap of gut microbiota between pSS and control groups. (**B**) Chao1 richness index. (**C**) Shannon diversity index. (**D**) PCoA plots based on unweighted Unifrac distances between the pSS and control groups. Key: blue dots, control group; green dots, pSS group; *, *P*<0.05.

A Venn diagram was used to visualize the composition of gut bacterial communities (**Fig 1A**). The numbers of genera in the pSS and control groups were 1,363 and 1,324, respectively. The total richness of genera in the two groups was 1,678. The number of genera shared by the two groups was 1,009, 60.13% of all observed genera. Next, we compared the alpha diversity in the pSS and control groups. The pSS group had higher community richness (Chao1: *P* = 0.0169; **Fig 1B**) and alpha diversity (Shannon index: *P* = 0.0448; **Fig 1C**) than the control group. Beta diversity based on an unweighted UniFrac PCoA showed the separate clustering of the pSS and control groups (Adonis test: *P* = 0.0010; **Fig 1D**), with principal components 1 and 2 accounting for 12.43% and 9.42% of the total variance, respectively.

### Composition of the gut microbiota of pSS patients and healthy controls

**Fig 2:**
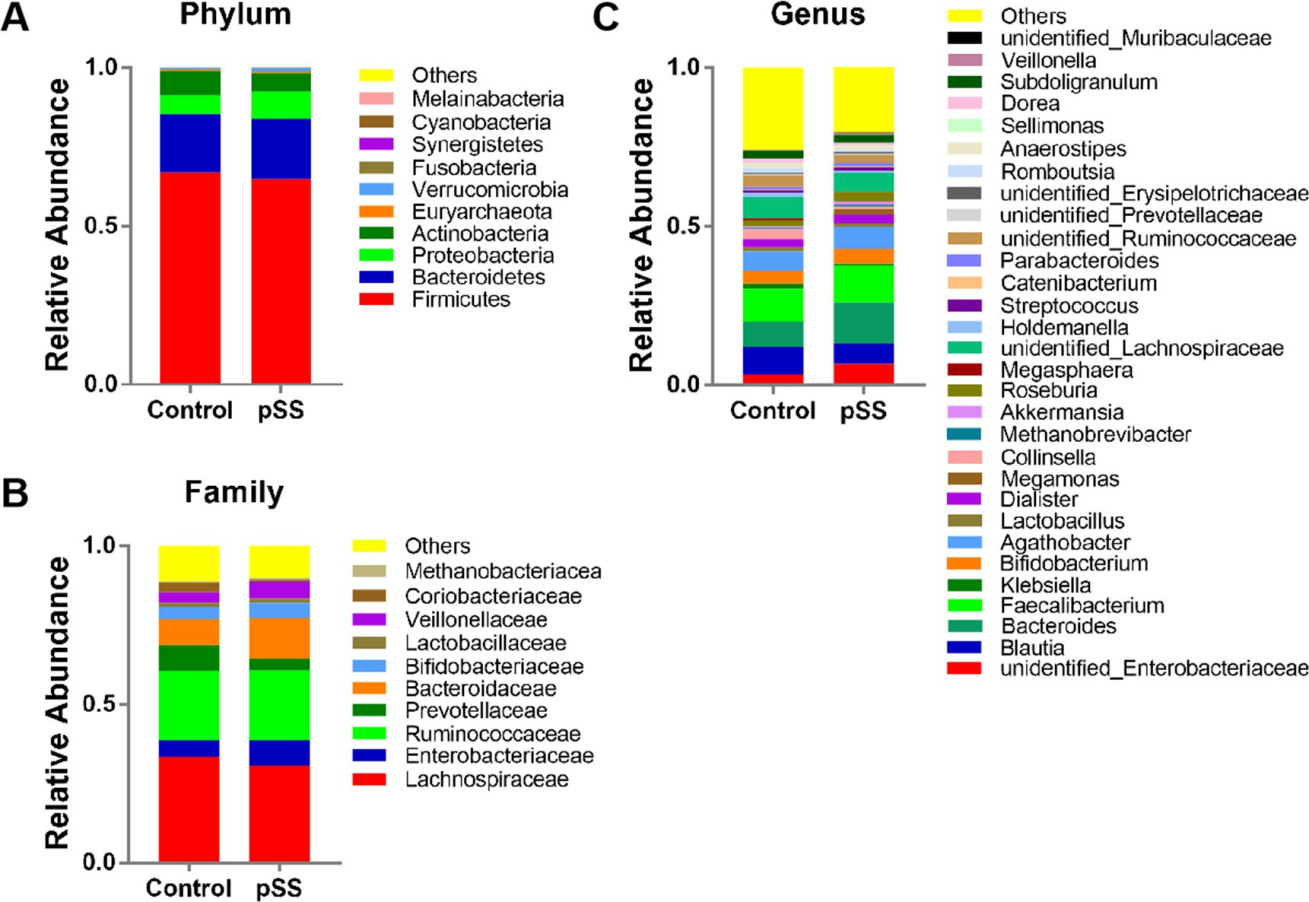
Composition of gut microbiota in pSS and control groups. (A) Phylum level. (B) Family level. (C) Genus level.

Four phyla (*Firmicutes*, *Bacteroidetes*, *Proteobacteria*, and *Actinobacteria*) accounted for >99% of the population in both of the pSS and control groups (**Fig 2A**). At the phylum level, we found a tendency for significantly more *Proteobacteria* in the pSS group (8.53%) than in the control group (5.82%; *P*>0.05). In addition, there were tendencies for significantly fewer *Firmicutes* (64.92% pSS vs. 66.90% control; *P*>0.05), *Actinobacteria* (5.89% pSS vs. 7.76% control; *P*>0.05), and *Firmicutes*/*Bacteroidetes* ratio (5.50 pSS vs. 7.09 control; *P*>0.05) in the pSS group than in the control group.

**Fig 3:**
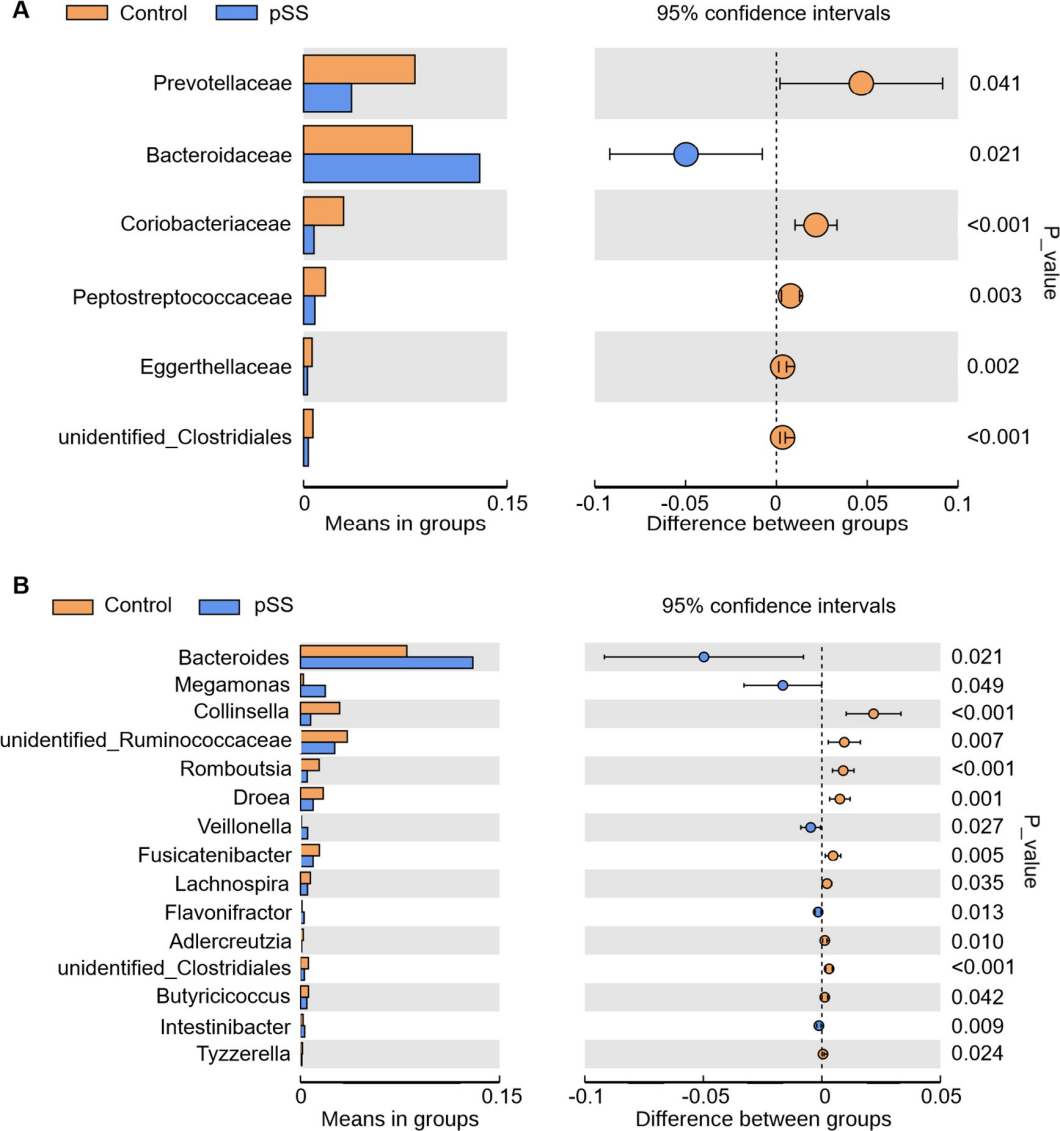
Abundances of gut microbiota in pSS and control groups. (A) Family level. (B) Genus level.

We further studied the compositional differences in gut microbiota at the family (**Fig 2B**) and genus (**Fig 2C**) levels between groups. Nine major families (abundance ≥0.01) were identified, among which *Prevotellaceae* was significantly decreased and *Bacteroidaceae* was significantly increased, in the pSS group compared to the control group (**Fig 3A**). In addition, we found significant decreases in *Coriobacteriaceae*, *Peptostreptococcaceae*, *Eggerthellaceae*, and unidentified_*Clostridiales* in the pSS group compared to the control group (**Fig 3A**). At the genus level, 15 genera showed significant compositional changes between pSS and control groups. We found significantly increased abundances of *Bacteroides*, *Megamonas*, *Veillonella*, *Flavonifractor*, and *Intestinibacter* in the pSS group compared to the control group. In addition, we found significantly decreased abundances of *Collinsella*, unidentified_*Ruminococcaceae*, *Romboutsia*, *Dorea*, *Fusicatenibacter*, *Lachnospira*, *Adlercreutzia*, unidentified_*Clostridiales*, *Butyricicoccus* and *Tyzzerella* in the pSS group compared to the control group (**Fig 3B**).

### Screening differentially abundant gut microbiota between pSS patients and healthy controls

**Fig 4:**
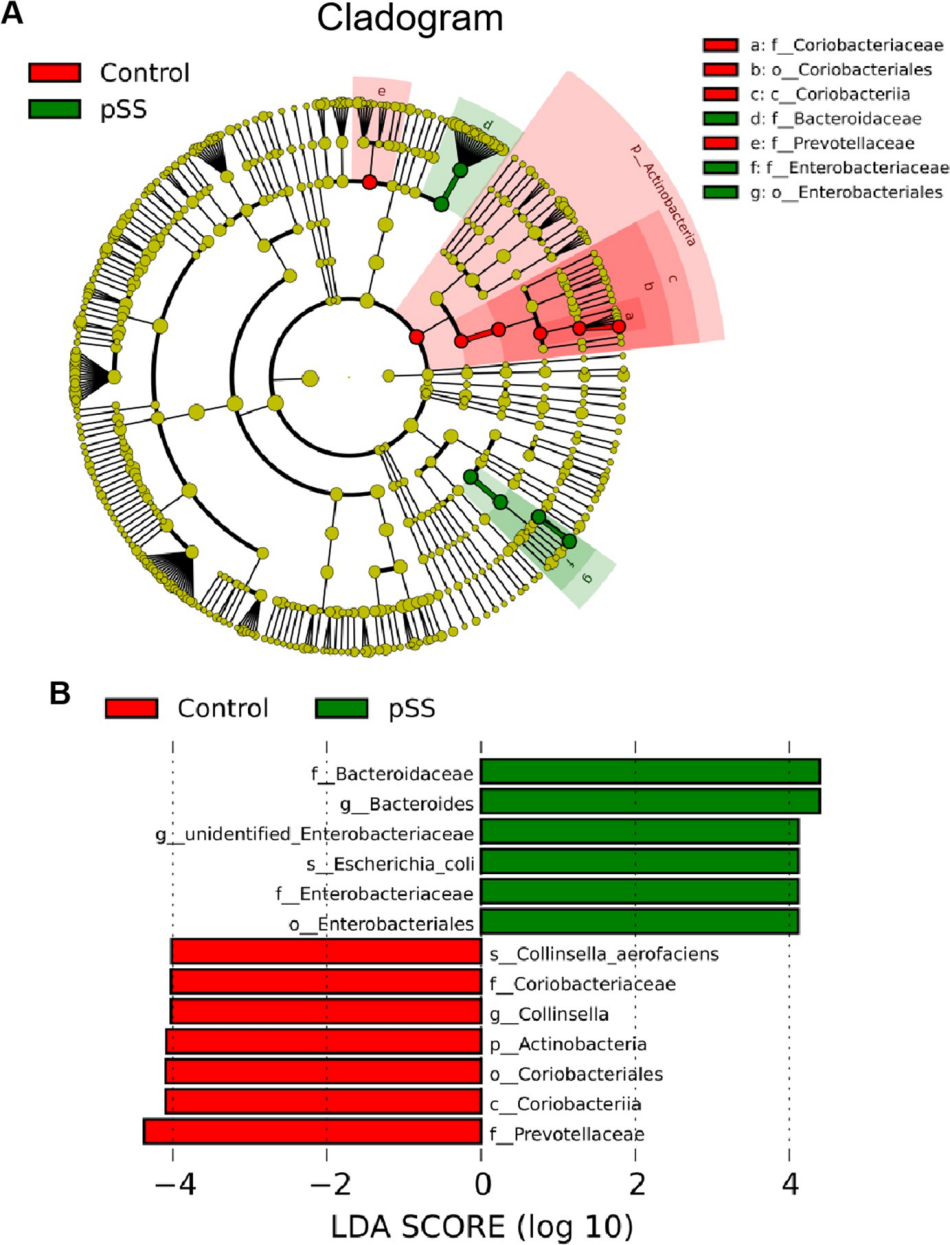
LDA effect size analysis. (**A**) Cladogram indicating the phylogenetic distribution of differential gut microbiota between the pSS and control groups. (**B**) The differential gut microbiota with an LDA Score >4 between pSS and control groups.

The LEfSe method, identified 13 significantly different components in the intestinal microbiota (LDA score>4) between the pSS and control groups. The results showed a discriminative association of order *Enterobacteriales*, families *Bacteroidaceae* and *Enterobacteriaceae*, genera *Bacteroides* and unidentified_*Enterobacteriaceae*, and species *Escherichia*_*coli* with the pSS group compared to the control group. In contrast, phylum *Actinobacteria*, class *Coriobacteriia*, order *Coriobacteriales*, families *Prevotellaceae* and *Coriobacteriaceae*, genus *Collinsella*, and species *Collinsella*_aerofaciens were significantly associated with the control group compared to the pSS group (**Fig 4**).

### Correlations between clinical characteristics and gut microbiota in pSS patients

**Fig 5:**
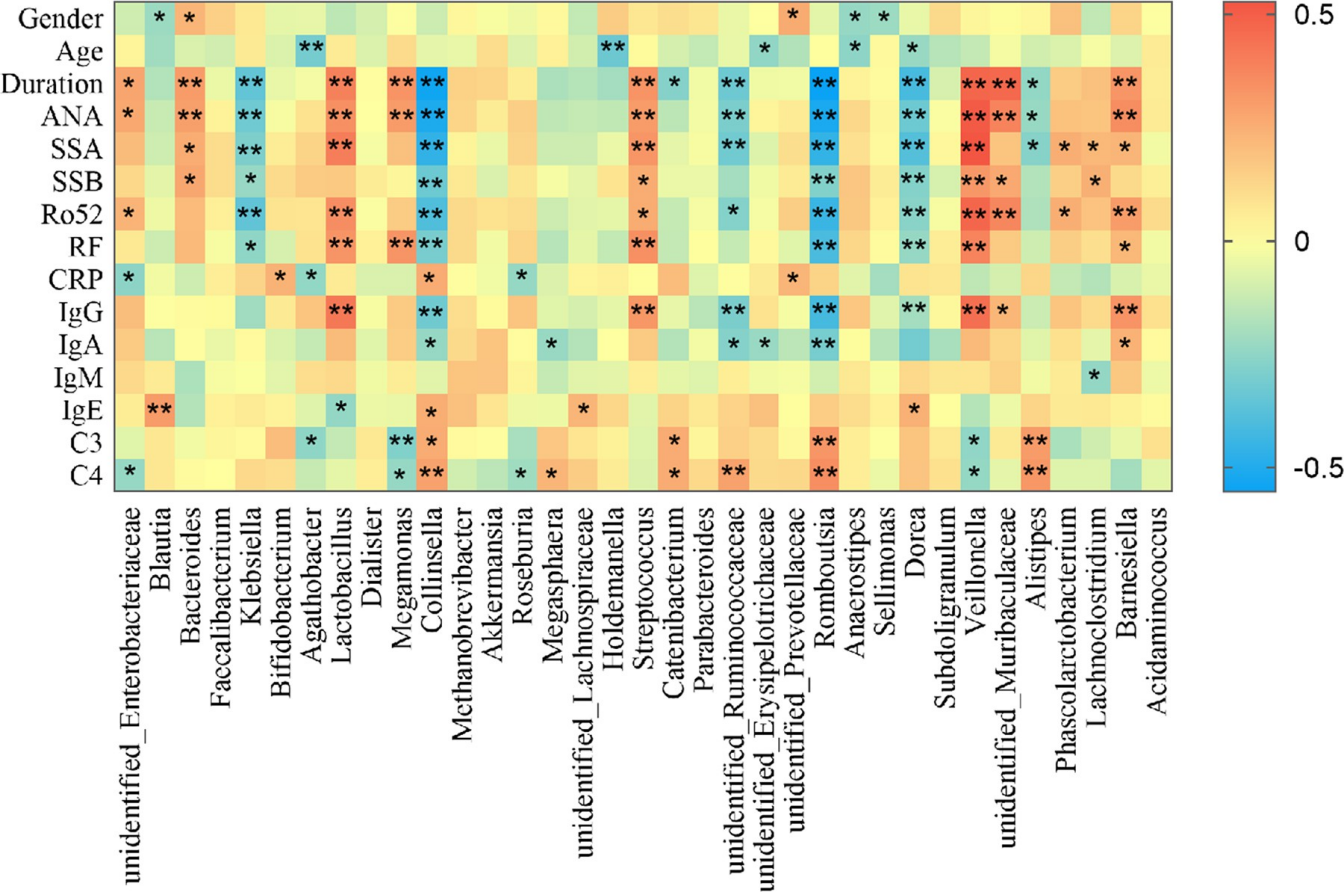
Heatmap of Spearman’s correlations between intestinal microbiota composition and clinical characteristics. Key: Red, the highest score (correlation); blue, the lowest score (correlation); *, *P*<0.05; **, *P*<0.01.

We analyzed correlations between intestinal microbiota composition at the genus level with several clinical characteristics in pSS patients (**Fig 5**). The genera *Bacteroides* and unidentified_*Prevotellaceae* were positively correlated with being female. The abundances of unidentified_*Enterobacteriaceae*, *Bacteroides*, *Lactobacillus*, *Megamonas*, *Streptococcus*, *Veillonella*, unidentified_*Muribaculaceae*, and *Barnesiella* were significantly positively correlated with disease duration, positive autoantibody percentages, and RF, IgG, and IgA levels, which were all significantly higher in pSS patients than in healthy controls. Moreover, these genera were negatively correlated with C3 levels, which were significantly lower in pSS patients than in healthy controls. In contrast, the abundances of genera *Klebsiella*, *Collinsella*, unidentified_*Ruminococcaceae*, *Romboutsia*, *Dorea*, and *Alistipes* were significantly negatively correlated with disease duration, positive autoantibody percentages, and RF, IgG, and IgA levels. Moreover, these genera were positively correlated with C3 levels.

## Discussion

pSS is characterized by dryness of the mouth and eyes. Several previous studies have reported dysbiotic salivary microbiota in pSS patients [17–23]. Intestinal dysbiosis has recently been considered a possible environmental influence in pSS etiology [1–3, 24]. To our knowledge, pSS patient characteristics in Northern China have never been reported. Here, we used high throughput sequencing to assesse 50 healthy individuals and 60 pSS patients to explore the intestinal microbial balance in pSS patients in Northern China. Taxonomic analyses showed that pSS patients had lower gut microbiota diversity than healthy controls. In addition, we found microbiota differences between pSS patients and healthy controls at the family and genus levels. We first investigated the relationship between gut microbiota and candidate biomarkers, finding that gut dysbiosis was associated with clinical and laboratory pSS markers, including increased positive autoantibody percentages and RF, IgG, and IgA levels and decreased C3 levels.

Intestinal dysbiosis is observed in autoimmune diseases, including IBD, SLE, and RA, and is associated with decreased bacterial diversity, increased pro-inflammatory bacteria, and decreased anti-inflammatory bacteria [25–27]. We found significant changes in alpha- and beta-diversities between pSS patients and healthy controls, suggesting that gut microbiota in the two groups differed significantly, consistent with a previous study [24].

At the phylum level, *Firmicutes*, *Bacteroidetes*, *Proteobacteria* and *Actinobacteria* were the dominant components of gut microbiota in pSS patients and healthy controls. The proportions of these four phyla did not differ significantly between groups. Nevertheless, their changing trends in pSS patients compared to healthy controls were similar to previous studies [17, 24].

At the family level, we found a significantly lower abundance of *Prevotellaceae* and higher abundance of *Bacteroidaceae*, contrary to prior data in pSS patients [24]. However, studies have shown that patients with multiple sclerosis may have a uniform decrease in *Prevotellaceae*, especially the genus *Prevotella* [28, 29]. Similarly, a study on Chinese RA patients showed them to have decreased *Prevotella* [30]. The *Prevotellaceae* family may be beneficial for enhancing the production of protective short chain fatty acids (SCFAs), such as butyrate and propionate, which are bacterial metabolites that expand gut regulatory T cells (Tregs) [28, 31]. In addition, Scher et al. reported that *Prevotella* abundance was negatively associated with *Bacteroides* [32]. The trend in *Bacteroides* abundance in our study was consistant with the findings of a previous study [24]. Importantly, we observed a significant positive correlation between autoantibody positivity in pSS patients and the relative abundance of *Bacteroides* in fecal samples. While *Bacteroides* species are commensal gut bacteria and are well-known for their increasing resistance to many antibiotics (33), some have been reported to be associated with the autoimmune diseases. Davis-Richardson et al. showed that *Bacteroides dorei* abundance correlated positively with future autoimmunity for type 1 diabetes [33]. In addition, *Bacteroides fragilis* has been identified as a potential gut pathobiont in autoimmune disease [34, 35]. The increase in *Bacteroides* in pSS patients observed in this study may enhance disease progression. The proliferation of families *Coriobacteriaceae* and *Eggerthellaceae* (phylum *Actinobacteria*) is reported to be triggered by polyphenols and fibers [36] and shows significant decreases in IBD patients compared to controls [26]. Furthermore, a decrease in the beneficial bacteria *Peptostreptococcaceae* (genus *Romboutsia*; phylum *Firmicutes*) was observed in irritable bowel syndrome patients [37].

At the genus level, we found a significant increase in the abundance of several gut opportunistic pathogens, *Bacteroides*, *Megamonas*, *Veillonella*, *Flavonifractor*, and *Intestinibacter* in pSS patients. The proportions of *Bacteroides*, *Megamonas*, and *Veillonella* showed significant positive correlations with clinical indicators in pSS patients. *Megamonas* and *Veillonella* were enriched in chronic hepatitis B patients [38], while *Flavonifractor* was abundant in patients with neuromyelitis optica spectrum disorders [39], and *Intestinibacter* was abundant in patients with Crohn’s disease [40]. In pSS patients, we also observed decreases in some probiotic genera: *Collinsella*, unidentified_*Ruminococcaceae*, *Romboutsia*, *Dorea*, *Fusicatenibacter*, *Lachnospira*, *Adlercreutzia*, unidentified_*Clostridiales*, *Butyricicoccus* and *Tyzzerella*. The proportions of *Collinsella*, unidentified_*Ruminococcaceae*, *Romboutsia*, and *Dorea* showed significant negative correlations with clinical indicators in pSS patients. *Dorea*, *Fusicatenibacter*, *Lachnospira*, and *Tyzzerella* are all Gram-positive bacteria and belong to the *Lachnospiraceae* family of *Firmicutes*. Zhou et al. [41] reported that the abundance of *Collinsella*, *Romboutsia*, *Dorea*, and *Fusicatenibacter* were significantly lower in allergic rhinitis patients than in healthy controls. In addition, the abundance of the *Dorea* genus was positively correlated with SCFA concentrations [41].

Among the above differential microbiota, *Veillonella* had the highest correlation with the positive rate of autoantibodies. In a recent systematic review, *Veillonella* was found to be significantly increased in three systemic autoimmune diseases, namely SLE, RA, and SS [42]. Therefore, *Veillonella* may be a factor related to intestinal dysbiosis in pSS patients in Northern China. However, more studies or different approaches are needed in the near future to find the clear connection between *Veillonella* and pSS patients. This study had several limitations. The results were not replicated in an independent cohort, did not distinguish between men and women due to the sex imbalance in our cohort, and were obtained with subjects from a single hospital. In addition, the results were compared to healthy controls only and not patients with symptoms of dryness without a pSS diagnosis. Nevertheless, this study confirmed intestinal dysbiosis in Northern Chinese pSS patients, which deserves further investigations.

## Conclusions

In summary, we confirmed that intestinal microbial diversity and composition in pSS patients differed from healthy controls in Northern China. Increased *Bacteroides*, *Megamonas*, *Veillonella*, *Flavonifractor*, and *Intestinibacter*, combined with decreases in *Collinsella*, unidentified_*Ruminococcaceae*, *Romboutsia*, *Dorea*, *Fusicatenibacter*, *Lachnospira*, *Adlercreutzia*, unidentified_*Clostridiales*, *Butyricicoccus* and *Tyzzerella* appear to be pSS characteristics in Northern Chinese patients. Spearman’s correlation analyses showed that *Bacteroides*, *Megamonas*, and *Veillonella* were positively correlated with clinical indicators in pSS patients, while *Collinsella*, unidentified_*Ruminococcaceae*, *Romboutsia*, and *Dorea* were negatively correlated. Among these differential microbiota, *Veillonella* may be a factor related to gut dysbiosis in pSS patients in Northern China. This study provides a theoretical basis for exploring novel diagnostic, prognostic, and treatment modalities in the new era of preventive, predictive and personalized medicine.

## Supporting information

S1 Table Demographic characteristics of the pSS patients and healthy controls. (DOCX)
