# Supplementary material for: Characteristics of gut microbiota in patients with primary Sjögren’s syndrome in Northern China
Source: PLoS One. 2022 Nov 10;17(11):e0277270. doi: 10.1371/journal.pone.0277270 (PMC9648750; doi:10.1371/journal.pone.0277270)
Supplement: S1 Table — (DOCX) [file pone.0277270.s001.docx]

**Journal name:** PLOS ONE

**Article ID:** PONE-D-22-09066

**Manuscript Title:** Characteristics of Gut Microbiota in Patients with Primary Sjögren's Syndrome in Northern China

**Authors:** Yuyuan Li, Zhi Li, Wenying Sun, Meiling Wang, Ming Li

**S1 Table. Demographic characteristics of the pSS patients and healthy controls**

|  | **pSS patients**  **(n=60)** | **Healthy Control**  **(n=50)** | **P value** |
| --- | --- | --- | --- |
| Marital status, married, n (%) | 54 (90%) | 44 (88%) | 0.957 |
| Smoking, n (%) | 2 (3.3%) | 5 (10%) | 0.157 |
| Education, mean±SD, years | 11.72±3.98 | 11.40±4.06 | 0.684 |
| Employment status, employed n (%) | 12 (20%) | 10 (20%) | 1 |
| Diet, n (%) | 0 | 0 | - |
| Vegetarians, n (%) | 2 (3.3%) | 2 (4%) | 0.854 |
| Medicine use, n (%) |  |  |  |
| Glucocorticoids, yes, n (%) | 25 (41.7) | - |  |
| Hydroxychloroquine, yes, n (%) | 52 (86.7) | - |  |
| Others, yes, n (%) | 55 (91.7) | - |  |
